# A case of picking calcified plaque in the common femoral artery with a long needle: the “calc-pick technique”

**DOI:** 10.1186/s42155-021-00263-1

**Published:** 2021-10-22

**Authors:** Yoshihiro Iwasaki, Shojiro Hirano, Atsushi Funatsu, Tomoko Kobayashi, Takanori Ikeda, Shigeru Nakamura

**Affiliations:** 1 Department of Cardiovascular Medicine, Toho University Graduate School of Medicine, Tokyo, Japan; 2 Department of Cardiovascular Medicine, Toho University Graduate School of Medicine, 1-banchi, 6-choume, Omori-nishi, Ota-ku, 143-8541 Tokyo, Japan; 3 Cardiovascular Center, Kyoto Katsura Hospital, Kyoto, Japan

**Keywords:** long needle puncture, severe calcified common femoral lesion, endovascular therapy

## Abstract

An 88-year-old man had intermittent claudication of his right leg. Angiography revealed severely calcified plaque in the common femoral artery. Endovascular treatment was performed by contralateral approach. We attempted to penetrate the center of the calcified plaque and perform balloon dilatation. However, a 0.014-inch stiff guidewire could not enter the center of the lesion. Thus, we used an inner cylinder of 15-cm 20G long needle directly through the retrograde femoral sheath and successfully introduced the guidewire into the calcified plaque. Crosser and balloon dilatation resulted in 50 % stenosis. To cross the center of calcified plaque, it is important to obtain sufficient lumen gain at the non-stenting zone.

## Background

Surgical endarterectomy is a first-line treatment for common femoral artery (CFA) disease (Katsushi et al. 2013) because CFA is a non-stenting zone. Recent advances in endovascular therapy (EVT) devices have reported effectiveness of EVT for CFA. Some reports have shown that balloon dilation enlarges the lumen by passing multiple wire routes within calcified plaques (Tan et al. 2016). However, it is not easy to pass the guidewire through hard plaque. We report a hard plaque penetration method using a 21G long needle.

## Case presentation

**Fig. 1:**
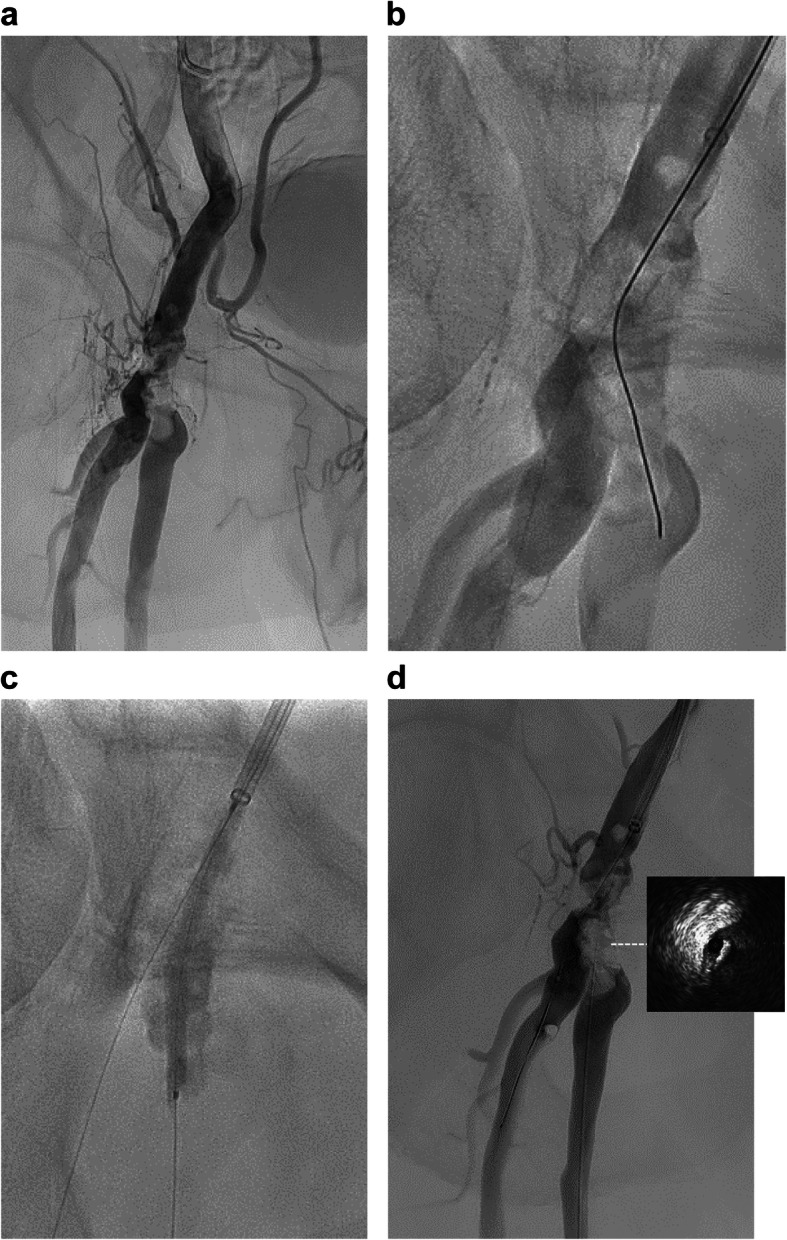
Angiography and intravascular ultrasound (IVUS) imaging. **A** Control angiography. **B** A 0.014-inch polymer jacket wire (Cruise, Asahi Intecc, Nagoya, Japan) was inserted into the deep femoral artery (DFA), and another tapered 45 g 0.014-inch wire (Jupitar Max, Boston Scientific, Natick, MA) was passed toward the SFA direction using a double lumen catheter (Crusade; Kaneka, Osaka, Japan). **C** A Crosser® catheter (FlowCardia Inc., Sunnyvale, CA) was passed six times toward the SFA direction, and the SFA was expanded with a 3 × 40 mm scoring balloon at 10 atm (NSE PTA; Nipro, Osaka, Japan). **D** Post-balloon angiography and intravascular ultrasound images

An 88-year-old man with diabetes mellitus presented with intermittent claudication (IC) of his right leg (Rutherford category III). His ankle brachial index (ABI) was 0.79 on the right side and 1.13 on the left side. Angiography revealed severe calcified stenosis in the CFA involving the proximal superior femoral artery (SFA) and the deep femoral artery (DFA) (Fig. 1A). Elective EVT for this severely calcified CFA lesion was selected to relieve symptoms.

**Fig. 2:**
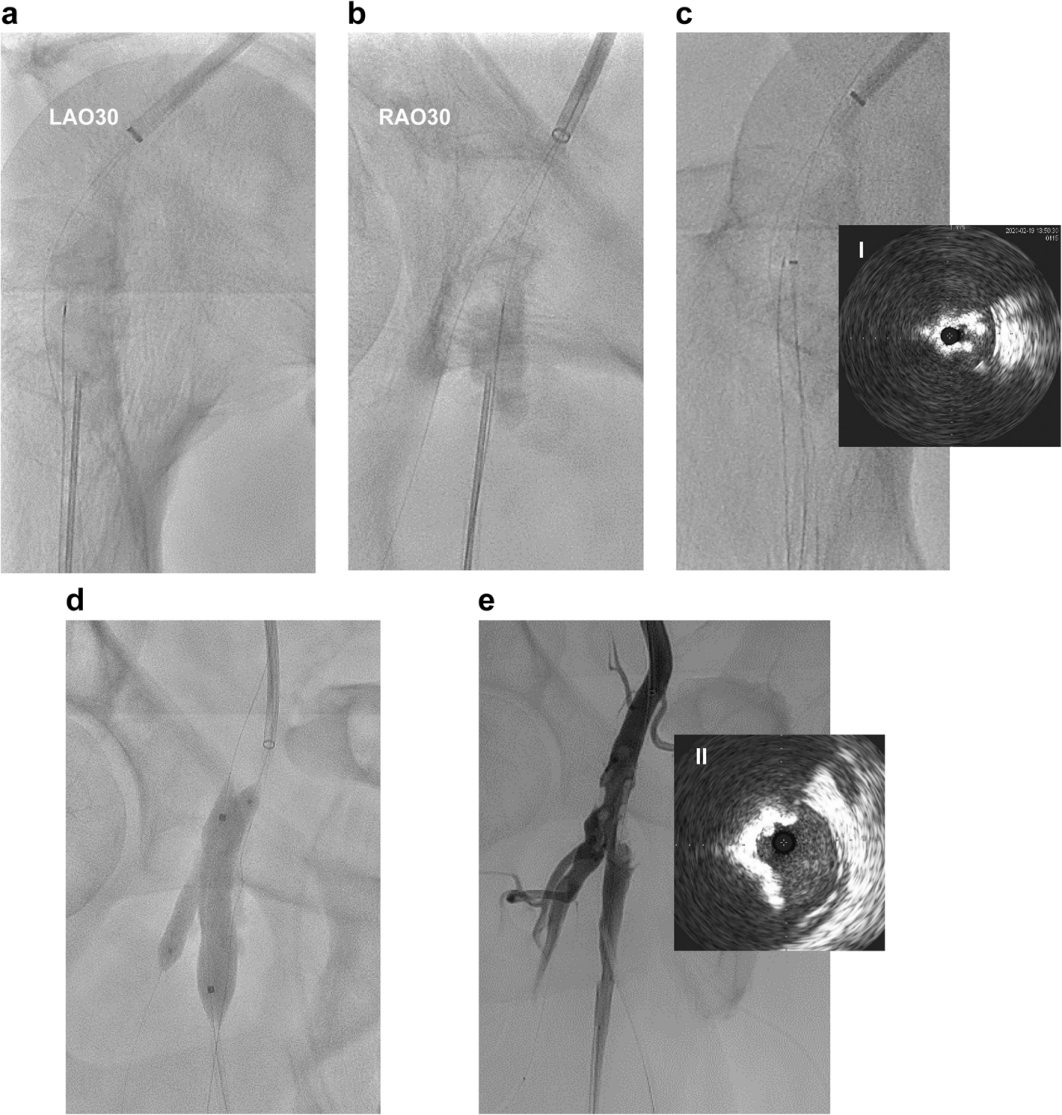
Angiography and intravascular ultrasound (IVUS) imaging. **A, B** Outer cylinder of 20G long needle (introducer needle, Medikit, Tokyo, Japan), the tip facing the center of the calcified plaque was checked by 2 perpendicular views in fluoroscopy. **C** Crosser. **C-I**) IVUS images showing the route created through the calcified plaque. **D** A kissing balloon inflation was performed with a 10 × 40 mm scoring balloon (NSE PTA; Nipro, Osaka, Japan) for SFA and with a 6 × 40 mm semi-compliant balloon (Sterling; Boston Scientific, Natick, MA) for DFA. **E**) final angiography. **E-II**) IVUS showing the lumen area gained

A 6-French guiding sheath (Destination, Terumo Interventional System, Somerset, NJ, USA) was inserted via the contralateral approach. A 0.014-inch polymer jacket wire (Cruise, Asahi Intecc, Nagoya, Japan) was advanced into the DFA, and a tapered 45 g 0.014-inch wire (Jupitar Max, Boston Scientific, Natick, MA) was passed into the SFA using a double lumen catheter (Crusade; Kaneka, Osaka, Japan) (Fig. 1B). A Crosser® catheter (FlowCardia Inc., Sunnyvale, CA, USA) was passed six times into the direction of the SFA, and the lumen was expanded with a 3 × 40 mm scoring balloon at 10 atm (NSE PTA; Nipro, Osaka, Japan) (Fig. 1C). Angiography showed insufficient lumen gain, and intravascular ultrasound (IVUS; Vision PV, Philips, Amsterdam, Holland) showed the wire route was at the edge of the vessel. Therefore, the Crosser and balloon dilatation procedures were not satisfactory (Fig. 1D). We unsuccessfully attempted to advance the guidewire into the center of the calcified plaque antegradely with a tapered 40-g 0.014-inch wire (VASSALLO 40, Filmeck, Nagoya, Japan). Given the severe calcification, we decided to make a direct puncture into the plaque using an inner cylinder of a 15-cm 20G long needle (introducer needle, Medikit, Tokyo, Japan). A 6-French sheath (Terumo Interventional System, Somerset, NJ, USA) was inserted retrogradely into the proximal SFA. After the needle was inserted through the sheath, we confirmed that the needle tip was facing the center of the calcified plaque from two directions (LAO30, RAO30) (Fig. 2-A and -B). We picked the calcified plaque with the needle to create a new route with gentle rotation. Then, while checking the silhouette of calcification from multiple directions, we introduced a tapered 40-g 0.014-inch wire (VASSALLO 40, Filmeck, Nagoya, Japan) through this needle. The wire was advanced relatively easily. Subsequently, a pull-throw guidewire position was established. Crosser was performed from an antegrade sheath. We confirmed that the guidewire had passed through the calcification using IVUS (Fig. 2-C1).

A kissing balloon dilatation was performed with a 10 × 40 mm scoring balloon (NSE PTA; Nipro, Osaka, Japan) on the SFA, and a 6 × 40 mm semi-compliant balloon (Sterling; Boston Scientific, Natick, MA) on the DFA (Fig. 2-D). The final angiogram showed that the stenosis still remained, but the blood flow was improved significantly, whereas IVUS showed a sufficient lumen area of 15.2 mm^2^ (Fig. 2-E②).

After the procedure, SFA sheath was removed. Hemostasis was achieved by manual compression and 6 × 40 mm semi-compliant balloon inflation at the puncture side.

IC was completely alleviated, and ABI was improved to 0.95 on the right side. At the 8-month follow-up, ABI remained stable, at 0.92 on the right side, without any other symptoms.

## Discussion

With the advancement of EVT devices and techniques, EVT treatment is applied to severely calcified CFA lesions. For example, Jet stream/pathway atherectomy (Boston Scientific, Natick, MA, USA) to reduce calcified plaque has been proven to be effective against severely calcified CFA lesions. (Manish et al. 2016).

However, the Crosser is the only available device for reducing calcified plaque in Japan. The Crosser may help to create a hole to modify the calcified plaque when the tip is facing it. Sometimes, it is impossible to cross the calcified plaque even with a 0.014-inch hard wire. EVT techniques for calcified lesion such as the Crossbow technique (Crosser® supported by bended 0.014 wire) have been reported (Tan et al. 2016). However, a route in the calcification cannot be made, and the effect is limited. In this case, we used the “calc-pick technique” to go through severely calcified plaque with the inner cylinder of a 20G long needle and cross the wire. The needle tip could not penetrate the calcified plaque by only 2 mm. However, the pushability of the wire was increased, and the wire could be passed through the new route into the calcified plaque. We encountered three cases of CFA with severe calcification at our hospital, all of which progressed without complications, and there were no claudication symptoms after 6 months.

**Fig. 3:**
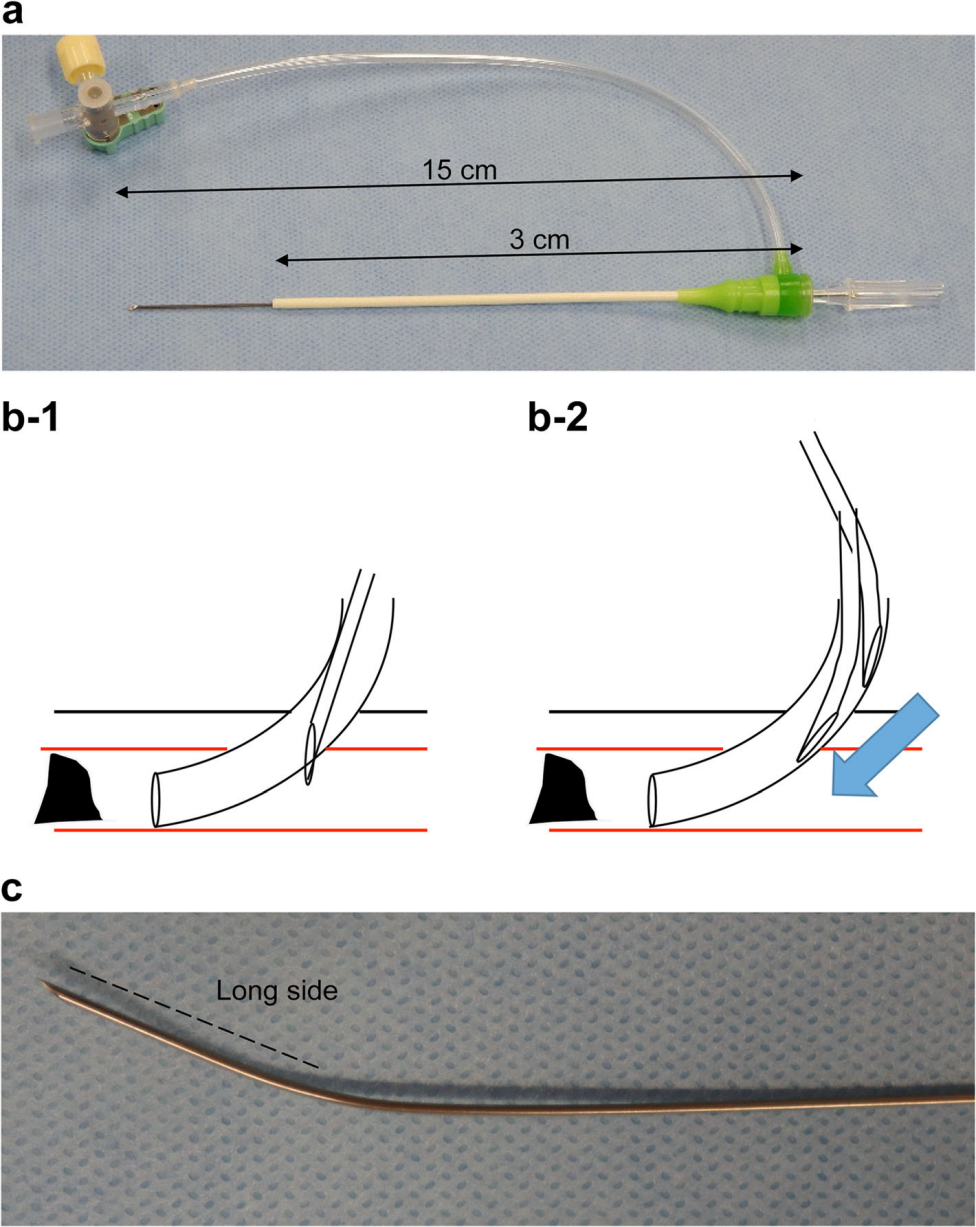
Needles used in the “calc-pick technique.” **A** Making a slight bend near the tip of needle at long axis side to improve tracking through the sheath; and **(B)** inner cylinder of 20G 12-cm long needle through the 6-French sheath

To apply the “calc-pick technique,” some important points should be noted. First, we selected a short 13-cm sheath to remove the needle tip. However, the needle advanced only 2 cm from the sheath (Fig. 3-A). Second, the SFA puncture point is located near the CFA calcified lesion, where the 15-cm needle could reach. Third, penetration of the sheath wall should be avoided. If the needle stays straight, it will penetrate the sheath wall (Fig. 3-B 1). Therefore, a slight bend was made near the needle tip. The needle angle depends on the retrogradely inserted sheath angle. (Figure 3-C). Furthermore, when introducing the needle to the artery, we checked that the needle did not penetrate the sheath wall using fluoroscopic guidance (Fig. 3- B 2). The needle position was checked in two perpendicular directions using fluoroscopy to confirm that it was in the center of the calcified plaque.

## Conclusions

Picking calcified plaque using a long needle (calc-pick technique) is a simple technique for EVT of severely calcified lesions in CFA. Thus, the calc-pick technique is very useful in performing EVT safely and effectively.

## Data Availability

Not applicable.

## Abbreviations

CFA: common femoral artery
SFA: superior femoral artery
DFA: deep femoral artery
IVUS: intravascular ultrasound
